# Supplementary material for: Single-molecule imaging reveals molecular coupling between transcription and DNA repair machinery in live cells
Source: Nat Commun. 2020 Mar 20;11:1478. doi: 10.1038/s41467-020-15182-3 (PMC7083905; doi:10.1038/s41467-020-15182-3)
Supplement: Supplementary file 1 — Supplementary Information [file 41467_2020_15182_MOESM1_ESM.pdf]

SUPPLEMENTARY INFORMATION

Single-molecule imaging reveals molecular coupling between transcription and DNA repair machinery in live cells

Han Ngoc Ho[1,2], Antoine M. van Oijen[1,2], Harshad Ghodke[1,2]

[1]Molecular Horizons and School of Chemistry and Molecular Bioscience, University of Wollongong, Wollongong, Australia

[2] Illawarra Health and Medical Research Institute, Wollongong, Australia

Correspondence and requests for materials should be addressed to H.G. (email: harshad@uow.edu.au)

Supplementary Figures

Supplementary Figure 1: The $k_{eff}\tau_{tl}$ plot for *mfd-YPet ΔuvrB* (solid line) indicates a single slowly dissociating species. For comparison, two simulated $k_{eff}\tau_{tl}$ plots describing a single dissociating species with k_{off} of 3.5x10^{-2} s^{-1} (τ_1 = 29 s; blue dashed curve) or 7x10^{-3} s^{-1} (τ_2 = 143 s; grey dashed curve) are provided. Shaded error bands represent standard deviations from ten bootstrapped samples. Simulations were performed with custom-written MATLAB codes as described in ref[1].

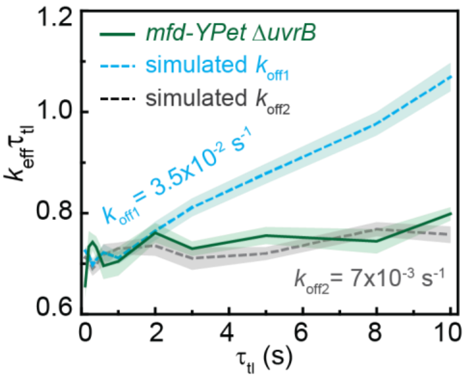

Supplementary Figure 2: Measurements of Mfd-YPet binding lifetimes in cells expressing the distal ATPase mutant UvrA(K646A).

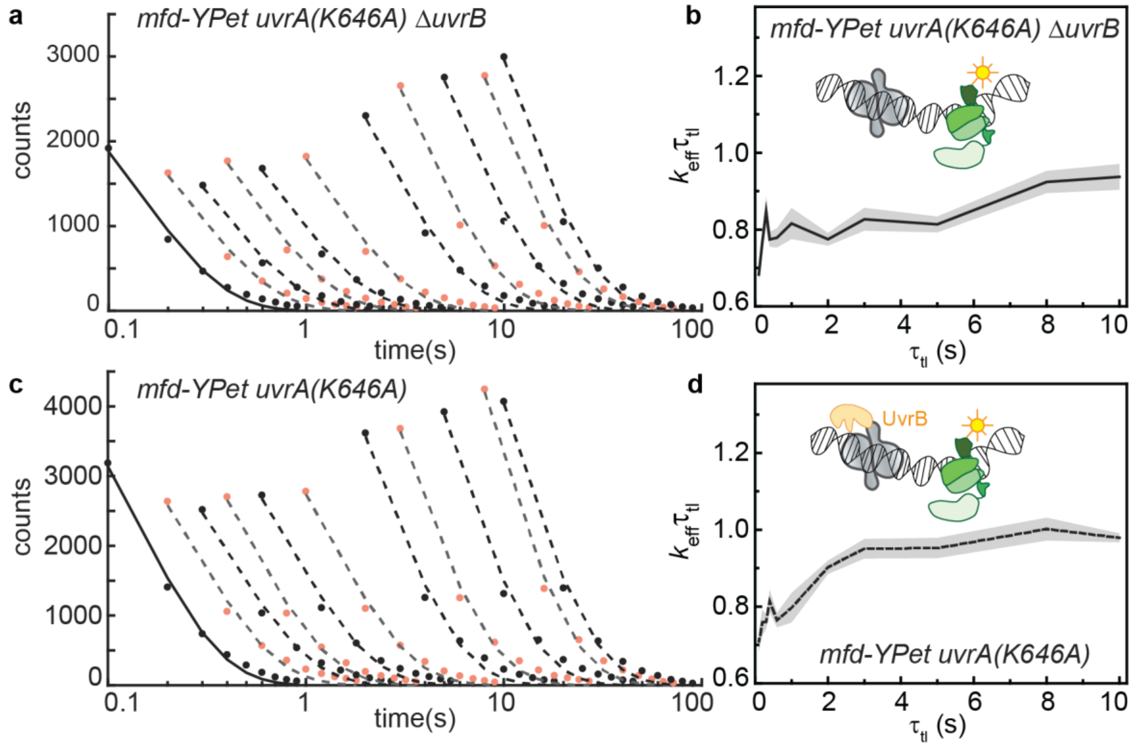

a. Cumulative residence time distributions (CRTDs, circles) obtained from interval imaging of Mfd-YPet in *mfd-YPet uvrA(K646A) ΔuvrB* cells. Lines are mono-exponential fits to CRTDs.

b. The $k_{eff}\tau_{tl}$ plot obtained from fitting CRTDs of Mfd-YPet in *mfd-YPet uvrA(K646A) ΔuvrB* cells. Cartoon (inset) illustrates the inability of UvrA(K646A) (grey) to interact with Mfd-YPet (green).

c. CRTDs (circles) obtained from interval imaging of Mfd-YPet in *mfd-YPet uvrA(K646A)* cells. Lines are mono-exponential fits to CRTDs.

d. The $k_{eff}\tau_{tl}$ plot obtained from fitting CRTDs of Mfd-YPet in *mfd-YPet uvrA(K646A)* cells. Cartoon (inset) illustrates the inability of UvrA(K646A)-UvrB (grey and orange) complex to interact with Mfd-YPet (green).

Shaded error bands are standard deviations from ten bootstrapped samples.

Supplementary Figure 3: Measurements of Mfd-YPet binding lifetimes in cells expressing the proximal ATPase mutant UvrA(K37A).

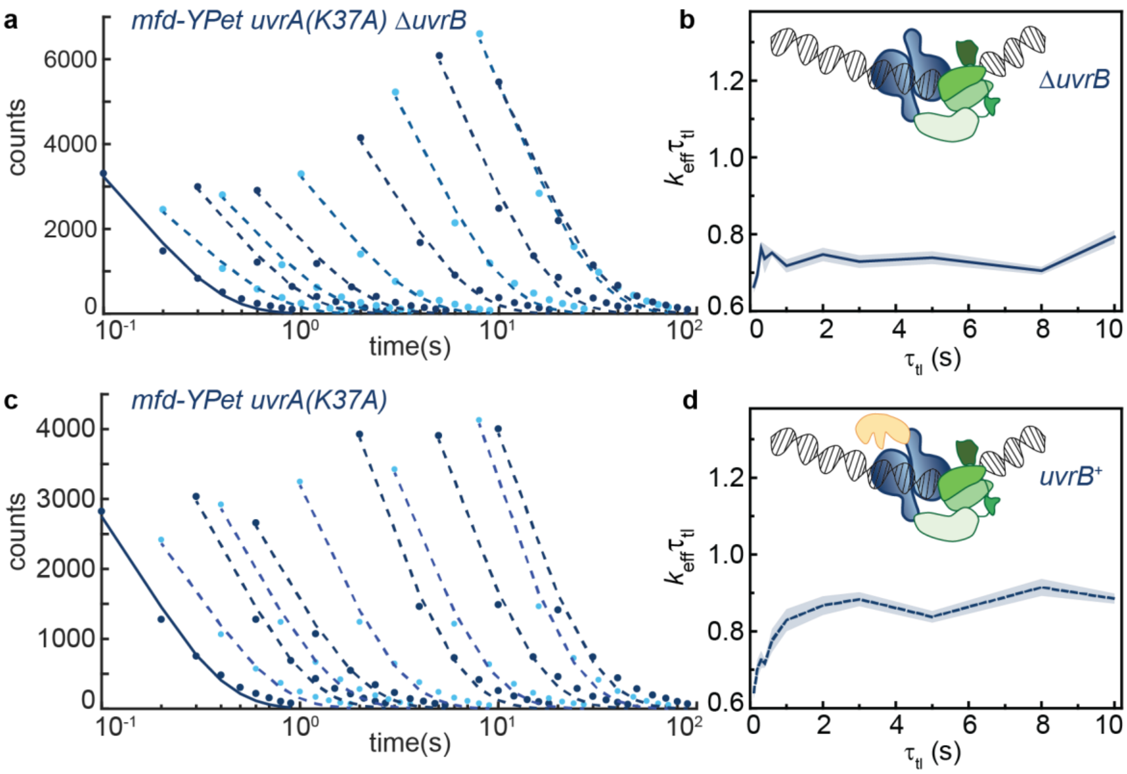

a. Cumulative residence time distributions (CRTDs, circles) obtained from interval imaging of Mfd-YPet in *mfd-YPet uvrA(K37A) ΔuvrB* cells. Lines are mono-exponential fits to CRTDs.

b. The $k_{eff}\tau_{tl}$ plot obtained from fitting CRTDs of Mfd-YPet in *mfd-YPet uvrA(K37A) ΔuvrB* cells. Cartoon (inset) illustrates the arrested complex formed by UvrA(K37A) (blue) and Mfd (green).

c. CRTDs (circles) obtained from interval imaging of Mfd-YPet in *mfd-YPet uvrA(K37A)* cells. Lines are mono-exponential fits to CRTDs.

d. The $k_{eff}\tau_{tl}$ plot obtained from fitting CRTDs of Mfd-YPet in *mfd-YPet uvrA(K37A)* cells. Cartoon (inset) illustrates the impaired handoff complex formed by UvrB (orange), UvrA(K37A) (blue) and Mfd (green).

Shaded error bands are standard deviations from ten bootstrapped samples.

Supplementary Figure 4: Measurements of Mfd-YPet binding lifetimes in cells expressing mutant UvrB deficient in DNA loading or over-expressing wild-type UvrB.

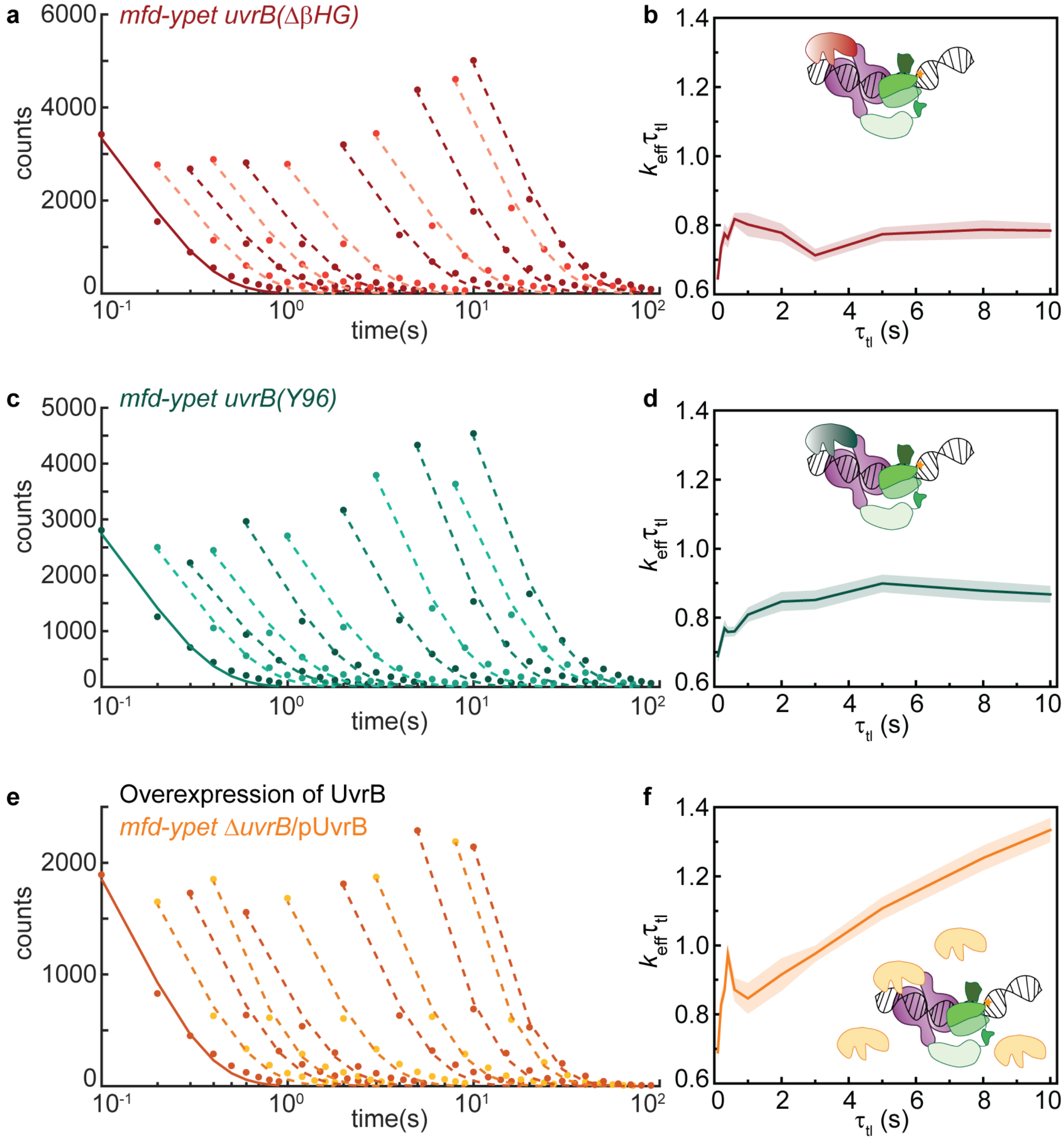

a. CRTDs (circles) obtained from interval imaging of Mfd-YPet in *mfd-YPet uvrB(*$\Delta\beta$*HG)* cells. Lines are mono-exponential fits to CRTDs.

b. The $k_{eff}\tau_{tl}$ plot obtained from fitting CRTDs of Mfd-YPet in *mfd-YPet uvrB(*$\Delta\beta$*HG)* cells. Cartoon (inset) illustrates the impaired handoff complex formed by UvrB($\Delta\beta$HG) (red), UvrA (purple) and Mfd (green).

c. CRTDs (circles) obtained from interval imaging of Mfd-YPet in *mfd-YPet uvrB(Y96A)* cells. Lines are mono-exponential fits to CRTDs.

d. The $k_{eff}\tau_{tl}$ plot obtained from fitting CRTDs of Mfd-YPet in *mfd-YPet uvrB(Y96A)* cells. Cartoon (inset) illustrates the impaired handoff complex formed by UvrB(Y96A) (cyan), UvrA (purple) and Mfd (green).

e. CRTDs (circles) obtained from interval imaging of Mfd-YPet in *mfd-YPet* Δ*uvrB*/pUvrB cells, in which UvrB is over-expressed from the pUvrB plasmid. Lines are mono-exponential fits to CRTDs.

f. The $k_{eff}\tau_{tl}$ plot obtained from fitting CRTDs of Mfd-YPet in *mfd-YPet* Δ*uvrB*/pUvrB cells. Cartoon (inset) illustrates the facilitated dissociation of Mfd in the context of the UvrB-$UvrA_2$-Mfd handoff complex.

Shaded error bands are standard deviations from ten bootstrapped samples.

Supplementary Tables

Supplementary Table 1: Bacterial strains. All strains are derivatives of *E. coli* K-12 MG1655.

Strain/genotypes	Source/Technique
MG1655 *mfd-YPet*	This laboratory
uvrB::kanR	This study/ λ Red recombination
mfd-YPet ΔuvrB	This study/ P1 transduction
mfd-YPet ΔuvrB/pUvrB	This study
mfd-YPet uvrA(K646A)	This study/ CRISPR-Cas9 assisted λ Red recombination
mfd-YPet uvrA(K646A) uvrB::kanR	This study/ P1 transduction
mfd-YPet uvrA(K37A)	This study/ CRISPR-Cas9 assisted λ Red recombination
mfd-YPet uvrA(K37A) uvrB::kanR	This study/ P1 transduction
mfd-YPet uvrB(ΔβHG)	This study/ CRISPR-Cas9 assisted λ Red recombination
mfd-YPet uvrB(Y96A)	This study/ CRISPR-Cas9 assisted λ Red recombination

Supplementary Table 2: Oligonucleotides used for colony PCR, λ Red recombination and cloning.

Oligo names	Sequence
Colony PCR for Cas9 verification	
dCas9dL5_303_F	CAGACCGCCACAGTATCAAA
pCas9_6700_R	GGAAGGTATCCGACTGCTG
Cloning of pCRISPR variants	
pCRISPR_UvrA_K646A_S	AAA CGT TAA TCA GCG TCG ATT TAC G
pCRISPR_UvrA_K646A_AS	AAA ACG TAA ATC GAC GCT GAT TAA C
pCRISPR_UvrA_K37A_S	AAA CGT GAC CGG GCT TTC GGG TTC G
pCRISPR_UvrA_K37A_AS	AAA ACG AAC CCG AAA GCC CGG TCA C
pCRISPR_UvrB_Y96A_S	AAA CCC TAC TAC GAC TAC TAT CAG CG
pCRISPR_UvrB_Y96A_AS	AAA ACG CTG ATA GTA GTC GTA GTA GG
ssDNA oligos for recombineering	
UvrA_K646A_ ssDNA	GGC GTT GGG CAA TCG GGA ACA GTG TGT CGT TAA TCA GCG TCG ACG CAC CGG AAC CTG AAA CCC CGG TGA TGC AGG TAA ACA GAC CCA
UvrA_K37A_ ssDNA	GCT GCC CTT CGG CAT ATA AGG TGT CGA AAG CGA GCG AGG ACG CGC CGC TAC CCG AAA GCC CGG TCA CGA CAA TGA GCT TGT CGC GGG
UvrB_Y96A_ ssDNA	CAA TGA AAG TGT CGG AAC TCG GTA CAT AGG CTT CCG GCT GTG CGT AGT CGT AGT AGG AAA CGA AAT ATT CCA CCG CGT TTT CC
UvrB_ΔβHG_ ssDNA	CAA TAT GTT CGT TAA CCG AGG CAT CTT TCT CAA TGA AAG TGC CAT AAT AGT CGT AGT AGG AAA CGA AAT ATT CCA CCG CGT TTT CCG

Supplementary table 3: Sequence of *uvrB* used in cloning

uvrB
CACCGCAATATATTGAATTTGCGTCGACCTCGAGCCCGGTACCCGGAGTTTACGCTGTATCAGAA<br>ATATTATGGTGATGAACTGTTTTTTTATCCAGTATAATTTGTTGGCATAATTAAGTACGACGAGTA<br>AAATTACATACCTGCCCGCCCAACTCCTTCAGGTAGCGACTCATGAGTAAACCGTTCAAACTGAAT<br>TCCGCTTTTAAACCTTCTGGCGATCAGCCAGAGGCGATTCGACGTCTCGAAGAGGGGCTGGAAGA<br>TGGCCTGGCGCACCAGACGTTACTTGGCGTGACTGGCTCAGGGAAAACCTTCACCATTGCCAATG<br>TCATTGCTGACCTTCAGCGCCCAACCATGGTACTTGCGCCCAACAAAACGCTGGCGGCCCAGCTG<br>TATGGCGAAATGAAAGAGTTCTTCCCGGAAAACGCGGTGGAATATTTCGTTTCCTACTACGACTA<br>CTATCAGCCGGAAGCCTATGTACCGAGTTCCGACACTTTCATTGAGAAAGATGCCTCGGTTAACG<br>AACATATTGAGCAGATGCGTTTGTCCGCCACCAAAGCGATGCTGGAGCGGCGTGATGTGGTTGT<br>GGTGGCGTCTGTTTCCGCGATTTATGGTCTGGGCGATCCTGATTTATATCTCAAGATGATGCTCCA<br>TCTCACGGTCGGTATGATTATCGATCAGCGCGCGATTCTGCGCCGACTGGCGGAGCTGCAATACG<br>CTCGTAATGATCAAGCATTCCAGCGTGGTACTTTCCGCGTTCGTGGCGAGGTGATAGATATCTTCC<br>CGGCAGAATCGGATGACATTGCACTTCGCGTGGAACTGTTTGACGAGGAAGTGGAACGATTGTC<br>GTTATTTGACCCGCTGACCGGGCAGATTGTTTCCACTATTCCACGTTTTACCATCTACCCGAAAACG<br>CACTACGTCACACCGCGCGAGCGCATCGTACAGGCGATGGAGGAGATCAAAGAAGAGCTGGCC<br>GCCAGACGCAAAGTGCTGTTGGAAAACAACAAACTGCTGGAAGAGCAGCGGCTGACCCAGCGTA<br>CCCAGTTTGATCTGGAGATGATGAACGAGCTGGGCTACTGTTCGGGGATTGAAAACTACTCGCGC<br>TTCCTCTCCGGTCGTGGACCGGGTGAGCCACCGCCGACGCTGTTTGATTACCTGCCTGCCGATGG<br>GCTGCTGGTCGTCGATGAATCTCACGTCACCATTCCACAAATTGGCGGCATGTATCGCGGTGACC<br>GGGCGCGTAAAGAGACACTGGTGGAGTACGGCTTCCGCCTGCCATCAGCGCTGGATAACCGTCC<br>GCTTAAGTTTGAAGAGTTCGAAGCATTAGCGCCGCAAACCATCTATGTTTCGGCGACGCCGGGTA<br>ATTACGAGCTGGAAAAATCCGGCGGCGATGTGGTGGATCAGGTGGTGCGTCCAACCGGATTGCT<br>TGACCCGATTATCGAAGTGCGGCCGGTGGCGACACAGGTTGATGATCTTCTTTCGGAGATTCGTC<br>AGCGAGCGGCAATTAACGAACGCGTACTGGTCACCACACTGACCAAGCGGATGGCGGAAGATCT<br>TACCGAATATCTCGAAGAACATGGCGAGCGCGTGCGTTATCTTCACTCAGATATCGACACCGTCG<br>AACGTATGGAGATTATCCGCGACTTGCGTCTGGGTGAGTTCGACGTGCTGGTAGGGATCAACTTA<br>CTGCGCGAAGGTCTGGATATGCCGGAAGTGTCGCTGGTGGCGATCCTCGACGCTGACAAAGAAG<br>GCTTCCTGCGTTCCGAACGTTCGTTGATCCAGACCATTGGTCGTGCGGCACGTAACGTTAACGGT<br>AAAGCGATTCTCTACGGCGATAAGATCACCCCATCAATGGCGAAAGCGATTGGCGAAACCGAAC<br>GTCGCCGTGAGAAACAGCAGAAGTACAACGAGGAACACGGAATTACGCCGCAAGGCTTGAACAA<br>GAAAGTGGTCGATATCCTGGCGCTGGGGCAGAACATTGCCAAAACCAAAGCGAAGGGCAGAGG<br>AAAATCGCGCCCGATTGTTGAGCCGGATAATGTGCCGATGGATATGTCGCCTAAAGCGTTGCAGC<br>AGAAAATCCATGAGCTGGAAGGGTTGATGATGCAACACGCGCAGAATCTGGAGTTCGAAGAAGC<br>GGCGCAAATTCGTGACCAGTTGCATCAGCTGCGTGAGCTGTTTATCGCGGCATCGTAAAGGAATT<br>CCATATGGGATAACCCGGTAAGG

Supplementary Table 4: Global fitting outputs for measurements of lifetime of Mfd-YPet in various genetic backgrounds. Bootstrapped CRTDs were fitted two single- and bi-exponential models (model 1 and 2 respectively). Selection of model was carried out as in ref.[1]. The chosen model and fitting outcomes are highlighted. Errors are standard deviations from ten bootstrapped CRTDs.

Derivatives of *mfd-YPet*	Model	k_b ± Error (s^{-1})	τ_1 ± Error (s)	*B* ± Error (%)	τ_2 ± Error (s)
Δ*uvrB*	**1**	**7.1 ± 0.1**	**139 ± 20**	**100**	**-**
	2	6.2 ± 0.1	1000	41 ± 2	5.5 ± 0.4
uvrA(K646A) Δ*uvrB*	1	7.5 ± 0.1	54 ± 8	100	-
	**2**	**5.9 ± 0.1**	**26 ± 2**	**32 ± 2**	**1.1 ± 0.1**
uvrA(K646A)	**1**	**7.9 ± 0.1**	**37 ± 3**	**100**	**-**
	2	6.9 ± 0.1	1000	26 ± 2	5.4 ± 0.2
uvrA(K37A) Δ*uvrB*	**1**	**7.2 ± 0.1**	**304 ± 69**	**100**	**-**
	2	6.0 ± 0.1	1000	40 ± 2	4.7 ± 0.2
uvrA(K37A)	**1**	**7.5 ± 0.1**	**52 ± 4**	**100**	**-**
	2	6.3 ± 0.3	1000	28 ± 1	4 ± 1
uvrB(ΔβHG)	**1**	**7.4 ± 0.1**	**188 ± 46**	**100**	**-**
	2	5.9 ± 0.4	50 ± 18	31 ± 5	1.2 ± 0.4
uvrB(Y96A)	**1**	**7.7 ± 0.1**	**70 ± 12**	**100**	**-**
	2	6.6 ± 0.1	1000	30 ± 2	5.2 ± 0.2
Δ*uvrB* /pUvrB	1	8.1 ± 0.1	18.1 ± 0.9	100	-
	**2**	**5.9 ± 0.2**	**11.1 ± 0.7**	**26 ± 2**	**0.50 ± 0.08**

Supplementary Methods

Strain construction

Strains of *mfd-YPet* expressing mutants of UvrA and UvrB from the chromosome were constructed using scar-less CRISPR-Cas9 assisted λ Red recombination as previously described[2, 3]. Briefly, *mfd-YPet* electrocompetent cells were transformed with pKD46[4] and pCas9[2]. The transformants were plated on LB plate containing ampicillin (50 µg per mL) and chloramphenicol (25 µg per mL) and were incubated at 30 °C overnight. As pCas9 is prone to recombination events[2], the resulting colonies were screened with colony PCR to confirm the presence of the full-length Cas9 gene, using primers targeting upstream and downstream of the Cas9 gene in pCas9.

Next, *mfd-YPet* cells harbouring pKD46 and pCas9 (HH438) were made electro-competent. First, the cells were grown at 30 °C with shaking at 200 rpm in 50 mL of LB containing ampicillin (50 µg per mL) and chloramphenicol (25 µg per mL). In these cells, Cas9 was constitutively expressed. The expression of λ Red recombination proteins was induced with 0.2% L-arabinose (w/v) when cells reached a density around 0.4 (OD_{600}). When the optical density reached 0.8, cells were pelleted at 4 °C and washed twice with ice-cold water, with an additional wash using 10% glycerol. Finally, aliquots containing 40 µL of cells in microcentrifuge tubes were snap-frozen in liquid nitrogen and stored at -80 °C.

Cas9 endonuclease activities were targeted to the vicinity of the desired point mutations on the *E. coli* chromosome with the help of the guide RNAs. These guide RNAs were designed such that each contains a 20-nt complementary sequence to that on the *E. coli* chromosome 5' of the PAM sequence (5'-NGG). They were expressed from pCRISPR variants (Supplementary Table 2), which we created following protocols from ref.[2].

The point mutations were introduced by recombining the foreign single-stranded DNA (ssDNA) (Supplementary Table 2). The ssDNAs were 80- to 90-nt oligos flanked by about 40-nt of sequence homologies to the *E. coli* chromosome on both sides of the desired mutations. Base changes were selected to be within five bases from the PAM sequence. This region, termed the seed region, is the most critical to Cas9 binding and disruption in this area ensure cells harbouring the point mutations are not targeted for subsequent cutting by Cas9.

Aliquots of cells expressing pCas9 and λ Red recombination proteins were transformed with 30 ng of pCRISPR variant plasmid and 500 ng of ssDNA. Positives were selected on LB plates containing 50 µg per mL of kanamycin and 25 µg per mL of chloramphenicol at 37 °C, and were screened by colony PCR, and the promoter and gene sequences were verified. Curing of pCRISPR variant plasmids was performed by propagating cells on LB plates for a week at 42 °C. This is critical for subsequent rounds of genome editing.

Supplementary References

1. Ho HN, Zalami D, Kohler J, van Oijen AM, Ghodke H. Identification of Multiple Kinetic Populations of DNA-Binding Proteins in Live Cells. *Biophys J* **117**, 950-961 (2019).

2. Jiang W, Bikard D, Cox D, Zhang F, Marraffini LA. RNA-guided editing of bacterial genomes using CRISPR-Cas systems. *Nat Biotechnol* **31**, 233-239 (2013).

3. Pyne ME, Moo-Young M, Chung DA, Chou CP. Coupling the CRISPR/Cas9 System with Lambda Red Recombineering Enables Simplified Chromosomal Gene Replacement in *Escherichia coli*. *Appl Environ Microbiol* **81**, 5103-5114 (2015).

4. Datsenko KA, Wanner BL. One-step inactivation of chromosomal genes in *Escherichia coli* K-12 using PCR products. *Proc Natl Acad Sci U S A* **97**, 6640-6645 (2000).
