# Supplementary material for: Single-molecule imaging reveals molecular coupling between transcription and DNA repair machinery in live cells
Source: Nat Commun. 2020 Mar 20;11:1478. doi: 10.1038/s41467-020-15182-3 (PMC7083905; doi:10.1038/s41467-020-15182-3)
Supplement: Supplementary file 3 — Description of Additional Supplementary Files [file 41467_2020_15182_MOESM3_ESM.pdf]

Description of Additional Supplementary Files

File Name: Supplementary Movie 1

Description: Movie showing continuous acquisition of images of *mfd-YPet* *ΔuvrB* cells imaged using a 514 nm laser. Integration time is 100 ms. Scale bar is 5 μm. Mfd-YPet in these cells exhibits both diffusive behavior (contributing to broad cytosolic fluorescence) and binding to RNAP resulting in distinct foci.
